# Sero-negative celiac disease with dermatitis herpetiformes: a case report

**DOI:** 10.1186/1757-1626-2-7512

**Published:** 2009-05-18

**Authors:** Mehreen Adhi, Asma Farooq, Syed Ali Hamid, Rabia Hasan, Salman Mamji, Akhtar Ali Baloch

**Affiliations:** 1 Department of Surgery, The Aga Khan University Hospital (Stadium Road)Karachi (74800)Pakistan; 2 Department of Medicine, The Civil Hospital (Baba-e-Urdu Road)Karachi (74400)Pakistan

## Abstract

**Introduction:**

We report a case of sero-negative celiac disease in Pakistan.

**Case presentation:**

A 20-year-old female presented with papulovesicular rash for 15 years, diarrhea for 8 years, spasms of hands and twitching of face for 4-5 months. She had mild anemia, low vitamin-D3 and serum calcium. On exclusion of other causes of malabsorption, anti-tissue transglutaminase antibodies (immunoglobulin-A & immunoglobulin-G), anti-endomysial antibodies, total immunoglobulin-A levels and skin biopsy were performed, which were normal. Intestinal biopsy revealed subtotal villous atrophy. Patient was prescribed gluten-free diet, to which she responded with alleviation of symptoms.

**Conclusion:**

Negative serology should not rule out celiac disease; intestinal biopsy should be performed if there is strong clinical suspicion.

## Introduction

Celiac disease (CD) is a disease entity characterized by damage of the small intestinal mucosa caused by the gluten contained in wheat and similar alcohol-soluble proteins of barley and rye, in genetically susceptible individuals [1]. The presence of gluten leads to self-perpetuating mucosal damage, whereas elimination of gluten results in full mucosal recovery [1]. The clinical manifestations of CD are protean in nature and vary markedly with the age of the patient, duration and extent of disease and presence of extra-intestinal pathological conditions [1]. In addition to the classical gastrointestinal form, a variety of other clinical manifestations of the disease have been described, including atypical and asymptomatic forms [1]. Thus, the diagnosis of CD can occasionally become extremely challenging [1]. The presence of Marsh 3 lesion (villous atrophy) on intestinal biopsy together with a positive antibody profile is currently internationally accepted as celiac disease [2,3] however, a European multicenter series reported antibody-negative celiac disease accounting for 6.4% of all celiac disease cases [4]. We present a case of a serology-negative celiac disease in Pakistan in a young woman, in whom the diagnosis would have been missed, had there not been a strong clinical suspicion.

## Case presentation

A 20-year-old unmarried female student, resident of a slum area of Karachi, presented in July 2008 in the out-patients clinic of a government-run tertiary-care hospital in Karachi, Pakistan, with complains of rash for the last 15 years and diarrhea (on and off) for the last 8 years, generalized weakness, fatigue, occasional spasm of both hands and twitching of the face for the last 4-5 months. The rash developed when she was 5 years of age, was initially papular, progressed to fluid-filled vesicles and was associated with severe itching. It occurred mostly along the back of her arms and trunk and at the front of the thighs, persisted for 1-2 months, and gradually improved to some extent by topical steroids and oral anti-histamines prescribed by a doctor in the local primary health care centre, only to recur after a period of 2-3 weeks.

At 11 years of age, patient developed symptoms of severe watery diarrhea, which followed a 1-2 week course, occurred 4-5 times in a day, unassociated with any particular food intake, settled down without treatment, only to recur after a symptom-free period of 2-3 days. There was no associated fever, nausea, dyspepsia, bloating, anorexia, weight loss, arthralgias or any neurological manifestations.

The patient denied any history of smoking or alcohol intake. Family history was also unremarkable.

The patient consulted various doctors in her locality who prescribed her multivitamins, calcium and multiple courses of a variety of antibiotics with no alleviation of symptoms. During the last 2 months, her symptoms increased in severity and she lost 4 kilograms weight.

On examination, patient had a thin, lean built (height: 5 feet, 2 inches and weight: 42 kg). She appeared pale and had papulovesicular rash; specially on the extensor surfaces of her thighs, legs, arms and trunk, and multiple hyperpigmented areas all over her body; residues of old healed lesions. Her systemic examination revealed coarse skin and hair and cheilosis around the mouth. Chvostek's and Trousseau's signs were also positive. On the basis of clinical features, patient was suspected to have a malabsorption syndrome.

Laboratory investigations revealed hemoglobin of 10.8 g/dl (hematocrit: 32%, MCV: 105cu-µm). Total leukocyte count (TLC), platelets, urea, creatinine, electrolytes, liver functions, serum proteins, prothrombin time, partial thromboplastin time, detailed reports of urine and stool, serum thyroid stimulating hormone (TSH) and parathyroid levels were all within normal ranges. In the light of decreased hemoglobin with high MCV, serum vitamin B12 and red cell folate levels were performed. Vitamin B12 levels were normal while red cell folate levels were at a lower normal range (200 ng/ml). Thus, folate deficiency was suspected to be the cause of the macrocytic blood picture. Serial serum calcium done in the last two months remained persistently low (6.9 mg/dl-7.5 mg/dl). Vitamin D3 levels were also low (28.8 n/ml). Chest X-ray was normal and X-rays of wrist and hands showed slight osteopenia. In the light of history and examination, complemented by papulovesicular skin lesions, a provisional diagnosis of celiac disease with dermatitis herpetiformis was made and anti-tissue transglutaminase (anti-TTG) antibodies (IgA and IgG) and anti-endomysial antibodies (anti-EMA) were performed. These were within the normal range. Serum total IgA levels were also normal.

Although the serology was negative, an endoscopy was performed on basis of a high index of suspicion and multiple biopsies were taken. Immunoflorescence biopsy of the skin lesions was also done. The patient was given a trial of gluten-free diet during her hospital stay. Interestingly, her diarrhea and skin lesions improved markedly within 1 week. The intestinal biopsy results later revealed subtotal villous atrophy with moderate chronic inflammation suggestive of celiac disease. The skin biopsy turned out to be normal, but in concert with Dermatology Department, the lesions were clinically diagnosed as dermatitis herpetiformes. The gene tests for HLA-DQ2 and HLA-DQ8 could not be performed due to unavailability of gene testing facilities. The histopathological confirmation, along with the response to gluten-free diet led to the final diagnosis of celiac disease with dermatitis herpetiformes.

Patient was counseled to continue life-long gluten-free diet, and was prescribed calcium and folate supplementation, multivitamins and minerals. Initially, she remained non-compliant and her symptoms continued, for which she also had to be re-admitted to the hospital. With repeated counseling, she understood the nature of her disease and gradually became compliant to gluten-free diet. With compliance, the diarrhea resolved completely, her hemoglobin rose to 11.9 gm/dl (MCV: 90 cu-µm), and calcium improved to 8.3 mg/dl. Nevertheless, due to the persistent skin lesions, she was prescribed Tablet Dapsone 50 mg/day. At the time of her last follow up, the skin lesions had also improved markedly and she reported considerable improvement in the quality of life.

## Discussion

The diagnosis of celiac disease can occasionally be extremely challenging owing to the variable clinical presentation. Although combination screening by anti-TTG and anti-EMA offer high sensitivity [5] seronegative celiac disease does occur [4,6] This is believed to be due to the fact that titers of anti-TTG and anti-EMA correlate with the degree of mucosal damage.[8-11]. Thus sensitivity is significantly lower in partial villous atrophy [9] as compared to full villous atrophy. Therefore, if the condition is suspected clinically and patient is deemed at high risk for celiac disease, even though serological tests are negative, intestinal biopsy should still be carried out [5,7]. Carroccio et al demonstrated that the culture medium of intestinal biopsy provides a higher sensitivity and diagnostic accuracy than serum assay for anti-EMA [12]. This may be a useful diagnostic tool for additional confirmation of the diagnosis of CD, particularly in patients in whom the serology is negative.

According to the European Society of Pediatric Gastroenterology and Nutrition (ESPGAN), the diagnosis of celiac disease does not require further confirmation if the initial diagnosis is based on appearance of flat intestinal mucosa with typical histological features of celiac disease on intestinal biopsy, and secondly on complete clinical remission on withdrawal of gluten from the diet [2]. The Interlaken procedure (gluten provocation test) may be required if there are doubts about the initial diagnosis, and especially in children under 2 years of age, where other causes of flat intestinal mucosa are much more prevalent [2]. In this case, although extensive workup was done to rule out other causes, and the intestinal biopsy findings and a complete clinical remission following gluten-free diet met the ESPGAN criteria for diagnosis of CD, but the fact that the serology for CD was negative, a gluten challenge test followed by a second intestinal biopsy could provide an additional confirmation in making a definitive CD diagnosis.

## Conclusion

Celiac disease is a potentially treatable condition, and the diagnosis can be easily missed if relied on serological markers alone. An intestinal biopsy should be performed if there is a strong clinical suspicion of CD.

**Figure 1.**
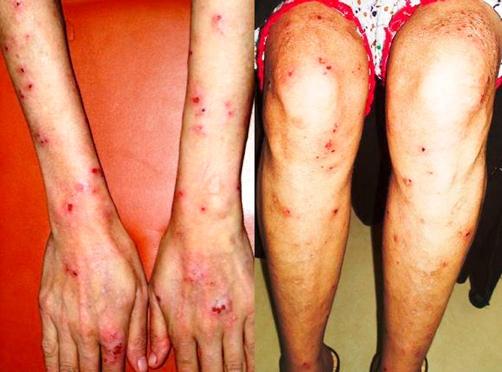
Photographic illustration of skin lesions on dorsum of hands, extensor surface of forearms, knees and extensor surface of the legs. Papulovesicular lesions spread out bilaterally on the extensor surface of the hands, forearms and leg. At this time, there is oozing of blood and vesicular fluid from the lesions, and no formed vesicles can be seen owing to the persistent itching experienced by the patient. These lesions improved markedly at the time of last follow-up after the patient was kept on a gluten-free diet and low dose dapsone therapy.

## List of abbreviations

Anti-TTG: Anti-tissue transglutaminase
Anti-EMA: Anti-endomysial antibodies
CD: Celiac Disease
ESPGAN: European Society of Pediatric Gastroenterology and Nutrition
IgA: Immunoglobulin A
IgG: Immunoglobulin G
MCV: Mean corpuscular volume
TLC: Total leukocyte count
TSH: Thyroid stimulating hormone
